# Children’s reliance on the non-verbal cues of a robot versus a human

**DOI:** 10.1371/journal.pone.0217833

**Published:** 2019-12-19

**Authors:** Josje Verhagen, Rianne van den Berghe, Ora Oudgenoeg-Paz, Aylin Küntay, Paul Leseman

**Affiliations:** 1 Utrecht University, Department of Special Education: Cognitive and Motor Disabilities, Heidelberglaan 1, CS Utrecht, the Netherlands; 2 University of Amsterdam, Amsterdam Center for Language and Communication, Spuistraat,VB Amsterdam, the Netherlands; 3 Koç University, Department of Psychology, Rumelifeneri Yolu, Sarıyer, Istanbul, Turkey; University Hospital of Tübingen, GERMANY

## Abstract

Robots are used for language tutoring increasingly often, and commonly programmed to display non-verbal communicative cues such as eye gaze and pointing during robot-child interactions. With a human speaker, children rely more strongly on non-verbal cues (pointing) than on verbal cues (labeling) if these cues are in conflict. However, we do not know how children weigh the non-verbal cues of a robot. Here, we assessed whether four- to six-year-old children (i) differed in their weighing of non-verbal cues (pointing, eye gaze) and verbal cues provided by a robot versus a human; (ii) weighed non-verbal cues differently depending on whether these contrasted with a novel or familiar label; and (iii) relied differently on a robot’s non-verbal cues depending on the degree to which they attributed human-like properties to the robot. The results showed that children generally followed pointing over labeling, in line with earlier research. Children did not rely more strongly on the non-verbal cues of a robot versus those of a human. Regarding pointing, children who perceived the robot as more human-like relied on pointing more strongly when it contrasted with a novel label versus a familiar label, but children who perceived the robot as less human-like did not show this difference. Regarding eye gaze, children relied more strongly on the gaze cue when it contrasted with a novel versus a familiar label, and no effect of anthropomorphism was found. Taken together, these results show no difference in the degree to which children rely on non-verbal cues of a robot versus those of a human and provide preliminary evidence that differences in anthropomorphism may interact with children’s reliance on a robot’s non-verbal behaviors.

## Introduction

Children are increasingly often exposed to new technologies in educational settings, such as applications on tablets and smartphones. One recent technology that has been employed for educational purposes involves social robots [1]. Social robots are specifically designed to communicate with people, either autonomously or semi-autonomously, following behavioral norms [2], and thus differ from robots used in factories that typically do not interact with people. Previous work on robot-assisted learning has investigated, amongst others, whether robots are effective in teaching language [3,4]. The common rationale in these studies is that robots offer advantages over human teachers and more traditional technologies, as they allow for student-paced and adaptive programs, but, at the same time, are embodied agents, which can interact with the social and physical world [1,3,4].

An often implicit assumption in robot-assisted language learning (RALL) studies is that learners can employ the non-verbal behaviors of a robot, such as eye gaze and pointing, for learning. Young children rely on non-verbal behaviors to learn new label-meaning mappings [5,6] and disambiguate between possible meanings of new words [7–9] if these are provided by a human. However, it is as yet unknown whether children rely to the same extent on non-verbal cues if these are provided by a robot. The primary aim of the current study is to investigate whether children rely on a robot’s pointing and eye gaze if these are contrasted with verbal labels to the same extent as on a human speaker’s pointing and eye gaze. A further aim of the study is to see whether children’s reliance on non-verbal cues of a robot is related to their perception of the robot as a human-like entity, that is, to the degree to which they anthropomorphize the robot. Investigating whether children rely on a robot’s non-verbal cues to the same degree as on those of a human is important for evaluating previous research findings in which social robots have been used as (language) tutors or teaching assistants (for a review, see [3]). In these studies, robots have typically been programmed to display non-verbal behaviors while interacting, such as looking at and tracing people’s faces, looking around, and gesturing [10–12]. The tacit assumption in these studies is that children (and adults) interacting with the robot pick up on these cues and use them for learning, as they would with a human teacher.

Previous research on language development has shown that young children do not only use a speaker’s verbal information to figure out the meaning of new words, but also rely on a speaker’s non-verbal communicative cues, in particular, on pointing gestures and eye gaze ([8,13,14], but see [15]. Specifically, young infants can use a speaker’s eye gaze to map a novel word to an object, even when this object is out of view [5,16] and learn a novel label for an object when they see a speaker gaze at an object, but not when they only hear a speaker’s voice [6].

In fact, a number of studies have demonstrated that children rely more strongly on a non-verbal cue than on a verbal cue in figuring out which object a speaker refers to. Grassmann and Tomasello [8], for example, administered a disambiguation task in which children were presented with two objects (e.g., a car and a novel object). The experimenter then verbally referred to the familiar object (“Give me the car”), while she pointed at the novel object, or vice versa (i.e., the experimenter asked for “modi”, while pointing at the car). Grasmann and Tomasello found that German two- and four-year-old children relied on pointing more strongly than on labeling in resolving this conflict, as children overwhelmingly handed the object pointed at to the experimenter. This preference for pointing was stronger when the experimenter used a novel label (e.g., “modi”) while pointing at a familiar object than when she used a familiar label (e.g., “car”) while pointing at an unfamiliar object. On the basis of these findings, the authors concluded that young children attribute more importance to socio-pragmatic cues than to verbal cues when resolving a referential conflict, especially so if they are uncertain about the meaning of a word (for replication studies, see [14,17,18]).

Children thus are willing to give up an important word learning heuristic–mutual exclusivity–in favour of a non-verbal cue. Mutual exclusivity is a word learning principle children use to figure out the meaning of new words. This principle bears on two important assumptions that children use in word learning: (i) two different words do not refer to the same referent, and (ii) two referents do not bear the same label. Thus, when presented with a novel label in the context of an unfamiliar and a familiar object, children assume that the novel label refers to the referent for which they do not have a verbal label yet [19,20].

To the best of our knowledge, only one previous study has compared how children use non-verbal cues for learning across a robot and a human speaker. In this study by Kory Westlund et al. [21], two- to five-year-old children needed to follow a speaker’s eye-gaze or bodily orientation to figure out the referent of a novel word. The authors found that children learned new label-referent mappings above chance level, irrespective of whether a robot or human adult administered the task. On the basis of these findings, they concluded that children can use eye gaze and bodily orientation to learn new words from a robot as well as from a human.

The results by Kory Westlund et al. leave open, however, how children weigh socio-pragmatic and verbal cues if both cues are in conflict. One possibility that cannot be ruled out on the basis of Kory Westlund et al.’s results is that children can use a robot’s non-verbal cues to figure out the meaning of a new word, but only in situations in which they cannot rely on verbal information instead. An outstanding question, therefore, is how children *weigh* a robot’s non-verbal cues as compared to those of a human: Do they also consider them to be the primary, most reliable cue for word meaning if they conflict with a verbal cue?

Another issue left open by Kory Westlund et al. is how children respond to *pointing* gestures by a robot. These authors examined two other types of non-verbal cues (i.e., eye-gaze and bodily orientation) rather than pointing gestures, as the robot in their study did not have arms. Pointing is much more salient than eye gaze, and has proved a much stronger cue than eye gaze in referential conflict tasks of the type discussed above [15]. Because of the potentially important role pointing can play in robot tutoring, it is worth investigating how children weigh the pointing gestures of a robot versus those of a human.

Finally, a question left unaddressed by Kory Westlund et al.’s study is whether there are individual differences among children in how strongly they rely on a robot’s non-verbal cues. People have a tendency to anthropomorphize robots, that is, to attribute human form, characteristics, and/or behaviors to robots [22]. This tendency results in people having more effective collaborations with robots and evaluating interactions with robots more positively [23–27]. Previous work has shown that five- to sixteen-year-old children may attribute mental states to robots, even when they are aware of the robot having sensors or being controlled by an adult [28]. Children may also attribute cognitive and affective beliefs, such as theory of mind and the ability to remember people, to robots [28,29]. Younger children are generally more likely than older children to show anthropomorphism [28–30], but there are substantial individual differences in the degree to which they anthropomorphize robots [28]). It is as yet an open question if these differences in anthropomorphism are related to children’s reliance on a robot’s non-verbal cues.

The current study addresses three questions:

1. How do children weigh non-verbal cues (i.e., pointing and eye gaze) and verbal cues (i.e., labeling) from a robot versus a human speaker?
2. Do children weigh such non-verbal cues differently depending on whether these are contrasted with a novel label or a familiar label? Do any effects of label familiarity differ between a robot and a human?
3. Do children rely differently on a robot’s non-verbal versus verbal cues depending on the degree to which they anthropomorphize the robot?

We report on two studies that were conducted to address these questions. In Study 1, we tested children’s reliance on pointing versus labeling. In Study 2, we tested children’s reliance on eye gaze versus labeling. In both studies, children’s following of the non-verbal cue versus the labeling cue was compared across two conditions: one in which a robot provided the cues and one in which a human adult provided these cues. In each study, non-verbal cues were contrasted with a verbal cue that either involved a familiar label (e.g., “car”) or a novel label (e.g., “modi”), following earlier work [8,14].

Regarding question 1 above, we hypothesized that children would rely more strongly on non-verbal cues as opposed to verbal cues if these were provided by the robot as compared to a human: Thus, they would be more inclined to give up on mutual exclusivity in the case of a robot, as they may assume the robot to possess different lexical knowledge than a human. Specifically, we hypothesized that children would find it more likely that the robot would use the word “modi” for the familiar object “car” or the word “car” for a novel object than a human speaker. This would lead them to rely less on the robot’s verbal labels, and more on its non-verbal behaviors instead, regardless of the type of non-verbal behavior (i.e., eye gaze vs. pointing). While we predicted the same pattern for both pointing and eye gaze (i.e., stronger reliance on these non-verbal behaviors of a robot than of a human), we anticipated children to follow pointing more often overall than eye gaze, because it is a more salient cue [15,31].

Regarding question 2, we predicted that children would show a smaller difference in their reliance on non-verbal cues between the two labeling conditions (i.e., whether the non-verbal cue was pitted against a novel or familiar label) if a robot–rather than a human–presented the task. With a human experimenter, children have been shown to be more reluctant to relax mutual exclusivity in the case of a familiar object than with a novel object. That is, they are less likely to assume that “car” refers to a novel object than to assume that “modi” is a special type of car, and consequently, rely less on pointing with a familiar label than with a novel label, as in earlier work [8,14,15]. However, with a robot, we hypothesized that children would be as likely to assume the robot to have a different referent for “car” or that, in the robot’s lexicon, “modi” is an existing word, and consequently, show a smaller difference in their reliance on pointing depending on whether a novel or familiar label was presented.

Regarding the third question, we predicted that children who perceived the robot as less human-like would follow the robot’s non-verbal behavior overall more strongly than children who perceived the robot as more human-like. Specifically, we hypothesized that children who considered the robot as not resembling a human would rely even less on its verbal labels–assuming that it would speak a different language or use words differently–and thus more on its non-verbal behaviors, than children who considered the robot as human-like.

## Study 1

In Study 1, we investigated children’s reliance on pointing versus a verbal label in a disambiguation task that was either administered by a social robot or a human speaker. We also assessed the degree to which children considered the robot to display typical human characteristics, through a questionnaire. As outlined above, we predicted that (i) children would follow pointing over labeling overall more strongly in the robot condition than in the human-speaker condition, (ii) children would show a stronger preference for pointing over labeling if a novel label was presented than when a familiar label was presented, but more strongly so in the human-speaker condition than in the robot condition, and (ii) children’s preference for pointing would be negatively affected by the degree to which they perceived the robot as human-like.

## Method

### Participants

Participants were 60 monolingual Dutch kindergartners (22 girls, 37%) with a mean age of 61.8 months (*SD* = 6.0, range = 50–74). Mean age was 62.6 (*SD* = 6.5) and 60.5 (*SD* = 5.6) months for boys and girls, respectively. Three additional children were excluded, since they did not complete the perception questionnaire. Children were recruited from various schools in the Netherlands, and written informed consent for each child was obtained from their parents prior to data collection. Parents filled out a questionnaire, which contained questions on home language background (to confirm that no other languages than Dutch were spoken at home) and parents’ highest attained educational level. Specifically, parents reported their highest attained educational level on a scale with 1 (primary school), 2 (secondary school), 3 (vocational training), 4 (higher professional education), and 5 (university) as its scale points (for similar assessments, see [32–34]). The mean was 4.06 (*SD* = 1.01), based on values averaged over children’s parents (data available for 59/60 families). Approval for the larger project that the current study was embedded in (i.e., L2TOR, see [12]) was obtained from the Ethical Committee of the Faculty of Social Sciences at Utrecht University, the Netherlands, under number FETC16-039.

### Materials

#### Disambiguation task

In this task, a referential conflict was created by pitting a non-verbal (pointing) cue and a verbal (labeling) cue against each other, following the tasks used in previous research [8,14]. The aim of the task was to assess which cue children relied on most to solve the conflict. Specifically, in the original version of the task [8,14], an experimenter showed two objects to the child, placed them on the table in between the experimenter and the child, and subsequently verbally requested one object, while pointing at the other. By seeing which object children selected–the object pointed at or the object labeled–the relative weight children attribute to both cues can be determined.

In our task, we made two changes to the original procedure used in earlier work. First, we used tablets displaying photographs of the objects instead of the actual objects, since the robot we used was not capable of placing real objects on the table. Following Kory Westlund et al. [21], we used two tablets, each displaying one of the photos, to make sure that there was a large enough spatial distance between the two pictures for children to identify which picture the robot pointed at. Photographs of the objects used in Verhagen et al. [14] were presented. A second change involved the ‘reward’. In earlier studies children were invited to play with a ‘chute’ through which they could slide the objects, after they had selected one of the objects, but since we used photographs of objects rather than physical objects, this was not possible. Therefore, after selecting a photo, children were shown a short video animation of the photographed object fading away from the tablet screen.

**Fig 1:**
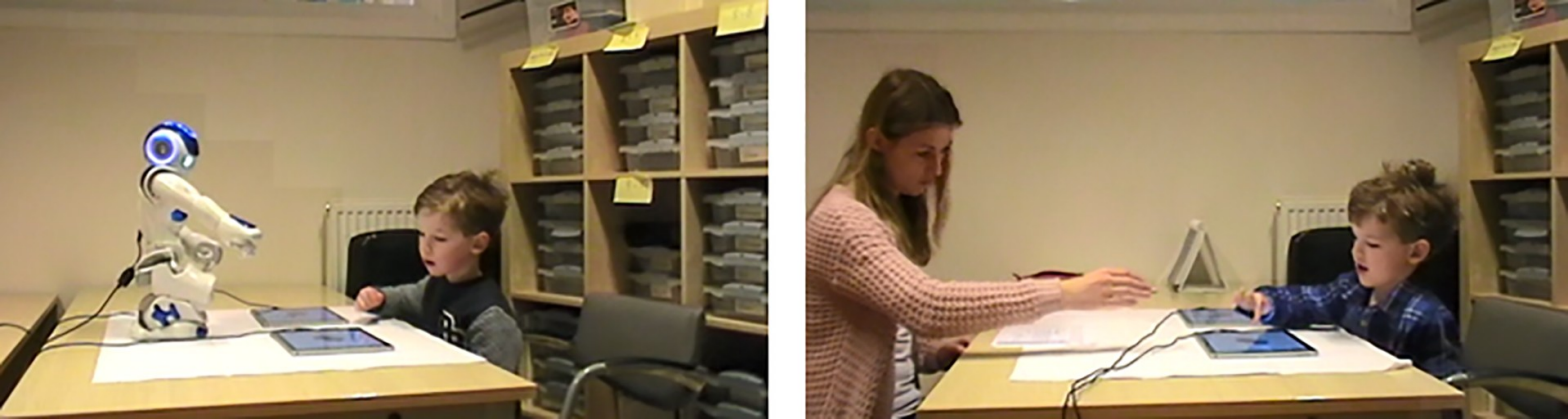
Child performing the task contrasting pointing and labeling with the robot and human experimenter. (Written informed consent for publication has been obtained from the child’s parents).

Two conditions were tested. First, in the ‘familiar-label condition’, the experimenter said the Dutch equivalent of the following instruction “Let’s play with the car. Tap on the car”. While producing this instruction, she pointed at the novel object. In the ‘novel-label’ condition, the experimenter said the Dutch equivalent of “Let’s play with the modi. Tap on the modi”. While producing this, she pointed at the familiar object (i.e., car). As in earlier work [8,14], ostensive pointing was used, as follows: The speaker looked at the child while pronouncing the Dutch equivalent of “Let’s play with the”. Then, the speaker pointed and gazed at the object while pronouncing the label for the first time (e.g., “modi”). Then, she looked back to the child while she kept pointing at the object and pronounced the Dutch equivalent of “Tap on the”. Then she looked back at the object again while she kept pointing at the object and produced the label for the second time. Since we aimed at keeping pointing consistent across the robot and the human speaker, and the robot could not move its fingers independently of each other, whole hand pointing was used by both the robot and the human experimenter (see Fig 1). The speaker’s pointing to the object (including looking back to the child while saying “Tap on the”) lasted about three seconds.

The experiment had a 2x2 design. Besides the ‘label’ condition (i.e., novel label vs. familiar label), there was a ‘speaker’ condition, as the task was either administered by a robot or by a human. Both the ‘label’ and ‘speaker’ conditions were administered within-subjects, so that each child was presented with the robot and the human, and performed both the familiar-label and novel-label trials. The two ‘speaker’ conditions were administered in two different sessions that were on average one week apart. The following factors were counterbalanced: the order of the ‘label’ conditions within the session, the order of the ‘speaker’ conditions across sessions, the placement of the images (left/right) vis-à-vis the child, the object labeled, the pairings of the novel and familiar images, the pairings of the novel words and objects, and the location of the image pointed at (left/right).

**Fig 2:**
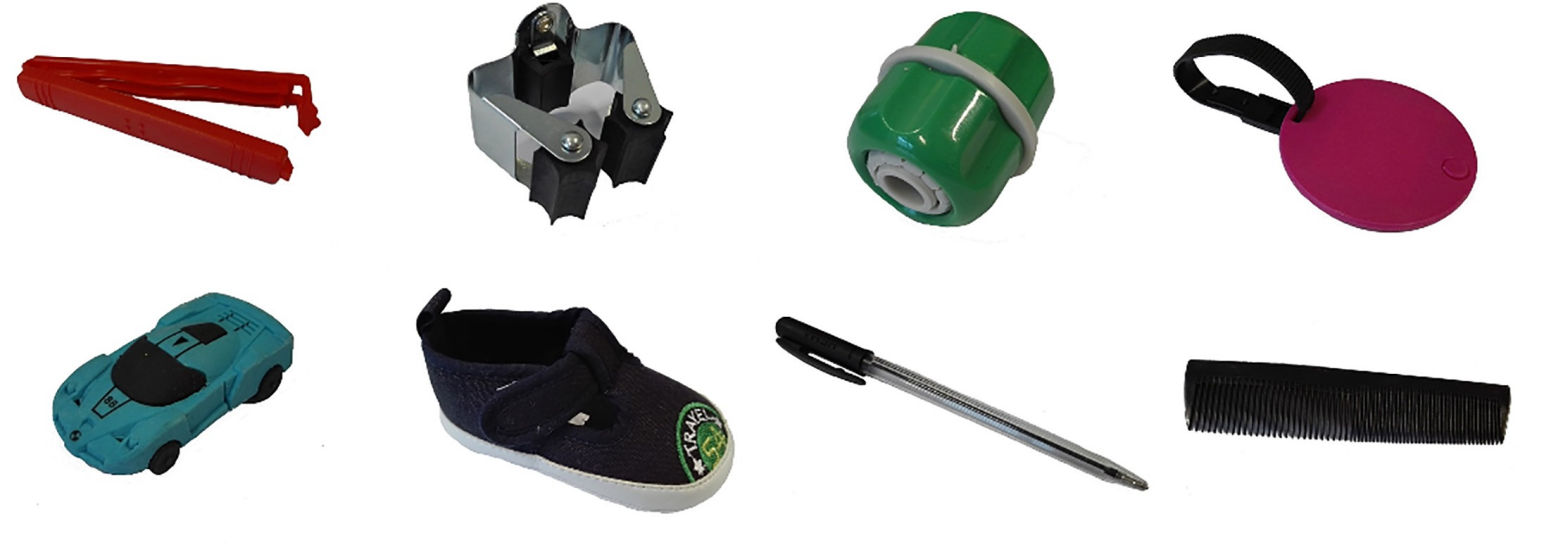
Images used in the disambiguation task. Novel objects in upper row; familiar objects in lower row.

There were four pairs of images in the familiar-label condition and four in the novel-label condition. In each pair, one of the images depicted a familiar object (i.e., a car, comb, pen, or shoe); the other depicted a novel object (i.e., a piece of garden hose, name tag, sealing clip or some building material)–see Fig 2. The novel labels (i.e., *toma*, *bafo*, *dofu*, *modi*) and familiar labels (i.e., the Dutch equivalents of *car*, *pen*, *shoe*, *comb*) were the same as in earlier studies [8,14]. The same items were administered once by the robot and once by the human speaker.

We used a Softbank Robotics NAO robot–a 58cm tall humanoid robot, which was controlled by an experimenter via a graphical user interface on a laptop computer located in the room, but not in direct sight of the child. The robot’s responses had been preprogrammed, such that its responses and behaviors were consistent for all children. During the disambiguation task, the robot was sitting in crouch position, see Fig 1. We ascertained that the verbal and non-verbal behaviors of the robot and the human experimenter were the same as much as possible. Specifically, the same instruction was provided prior to the task and the verbal and non-verbal behaviors during the trials were exactly the same across conditions. The robot was presented as “Robin the robot” to the children.

#### Perception questionnaire

We used a questionnaire adapted from Jipson and Gelman [35] to assess to what extent children perceived the robot as a human-like entity. The questionnaire was orally administered by the experimenter at the end of the session with the robot, and took about ten minutes to complete. It contained fourteen yes/no-questions, as well as, for each question, the follow-up question “Why?” or “Why not?”. Example questions are “Can Robin the robot see things?”, “Should Robin the robot eat?”, and “Can Robin the robot be happy?”. Scores were calculated as the total number of ‘yes’-answers for each child. Two items were removed from the scale (i.e., Can Robin the robot break? Is Robin the robot made by humans?), as children’s answers to the “Why (not)?” questions revealed a problem with the validity of these items. Specifically, for the item “Can Robin the robot break?”, children often answered ‘no’, referring to the firm materials it was made of rather than providing answers that indicated that they perceived of the robot as a machine rather than a human. Children’s ‘yes’-answers were summed, yielding a maximum score of twelve, with a higher score signaling higher anthropomorphism. The questionnaire had good internal consistency (Cronbach’s alpha = .80).

### Coding

Children’s responses were coded offline by trained assistants, based on video recordings. A response was coded as ‘point following’ or ‘label following’ when children chose the images pointed at or labeled, respectively. Furthermore, following Verhagen et al. [14], items were scored as ‘both items’ if a child selected both images, either simultaneously or successively. A subset of 10% randomly selected videos was coded by an additional assistant, yielding 100% agreement between coders.

### Procedure

All children participated in a group demonstration of the robot that took place in the children’s classroom prior to the first test session, in order to familiarize them with the robot and reduce any shyness or anxiety. In this familiarization session, the experimenter had the robot introduce itself as Robin the robot, perform a dance, and play a mimicking game with the children. The test sessions proper took place in a quiet room at children’s schools. Children were tested individually by trained experimenters. In the session with the human experimenter, children performed the trials of the novel-label condition and the familiar-label condition, in two separate blocks, in counterbalanced order. In between, they performed a vocabulary task not reported on in this paper. In the session with the robot, children were again administered the trials of the novel-label condition and the familiar-label condition in blocks, in counterbalanced order, with an intermediate task assessing compliance not reported on in this paper. This session was concluded with the perception questionnaire. The order of speakers across sessions was counterbalanced. Each session lasted 15–20 minutes. After each session, children received a small gift.

### Analyses

A series of *t*-tests were performed to see whether children followed pointing over labeling above chance level. Subsequently, generalized linear mixed-effect regression analyses were run in R version 3.4.1 [36], using the lme4 package [37], to address our research questions (see below). In all models, orthogonal sum-to-zero contrast coding was applied to our fixed effects [38]. To solve issues of non-convergence, we increased the number of iterations to 100,000 [39].

To address our first and second questions about possible differences between children’s reliance on pointing between a robot and a human speaker and between a novel label and a familiar verbal label, a model was run in which ‘speaker’ (robot vs. human) and ‘label’ (novel label vs. familiar label) were entered as fixed-effect factors. This model contained random intercepts ‘subjects’, and a by-subject random slope for ‘speaker’. Random intercepts could not be included for ‘items’, given that each item involved a label paired with different pairs of images (as the co-occurrence of labels and images was counterbalanced across participants). The dependent variable was children’s point following (0 = no point following, 1 = point following). Responses in which children selected no image (*n* = 1, < 1%) or both images (*n* = 5, < 1%) were very rare overall, and not taken into account. Additional models in which children’s age (in months), gender, and presentation order (whether they did the task with the robot first or last) were added as fixed-effect factors did not show effects of these factors, and–more importantly–did not yield different results for our variables of interest, and thus are not reported.

To address our third question regarding the possible effect of children’s perception of the robot on point following, the same model was run, with an additional fixed-effect factor ‘perception’ (i.e., children’s sum scores on the perception questionnaire), as well as interactions between this factor and ‘speaker’ and ‘label’. Since ‘perception’ was a continuous variable, it was centered around zero [38]. As above, additional models were run, controlling for age, gender, and presentation order, but since these did not yield effects and did not result in different outcomes for our variables of interest, these are not reported.

## Results

### Comparing children’s point following across conditions

Descriptive statistics are presented in Table 1 for children’s point following in the two label conditions (novel vs. familiar label) and the two speaker conditions (robot vs. human) separately.

**Table 1:** Mean proportions and standard deviations for children’s point following after hearing a novel label or familiar label from the robot or human.

	Novel label		Familiar label	
	M	(SD)	M	(SD)
Robot	0.67	(0.47)	0.69	(0.47)
Human	0.64	(0.48)	0.64	(0.48)

A series of *t*-tests showed that children’s following of the pointing gesture was significantly *above* chance in all conditions (i.e., *t*(54) = 2.869, *p* = .006, *d* = 0.39 for the robot using a novel label; *t*(54) = 3.125, *p* = .003, *d* = 0.43 for the robot using a familiar label; *t*(55) = 2.195, *p* = .032, *d* = 0.29 for the human speaker using a novel label; *t*(55) = 2.445, *p* = .018, *d* = 0.33 for the human speaker using a familiar label).

Results of a linear mixed-effect model on children’s point-following responses with ‘speaker’ (robot vs. human) and ‘label’ (novel vs. familiar) showed no effects (see Table 2).

**Table 2:** Results of a linear mixed-effect model on children’s point following with speaker (robot vs. human) and label (novel vs. familiar) as fixed factors.

	Estimate	SE	*z*	*p*
Intercept	2.313	0.740	3.127	.002
Speaker	-0.782	0.696	-1.124	.261
Label	-0.232	0.248	-0.937	.349
Speaker*Label	-0.242	0.497	-0.487	.626

### Investigating the effect of children’s perception of the robot

Children’s mean score on the perception questionnaire was 8.0 (*SD* = 3.0, max score = 12), indicating that, on average, children answered ‘yes’ to more than half of the questions on the robot’s human-like properties (e.g., being able to think, being happy). To investigate whether children’s perception of the robot was related to their point-following behavior across conditions, the above model was re-run, with children’s perception scores as an additional fixed-effect factor. The results of this model are presented in Table 3.

**Table 3:** Results of a linear mixed-effect model on children’s point following with speaker (robot vs. human), label (novel vs. familiar), and perception as fixed factors.

	Estimate	SE	*z*	*p*
Intercept	2.473	0.779	3.173	.002
Speaker	-1.020	0.748	-1.362	.173
Label	-0.031	0.271	-0.113	.910
Perception	0.303	0.228	1.324	.186
Speaker*Label	-0.412	0.537	-0.768	.443
Speaker*Perception	-0.200	0.185	-1.077	.281
Label*Perception	0.398	0.087	4.559	< .001
Speaker*Label*Perception	-0.295	0.173	-1.706	.088

**Fig 3:**
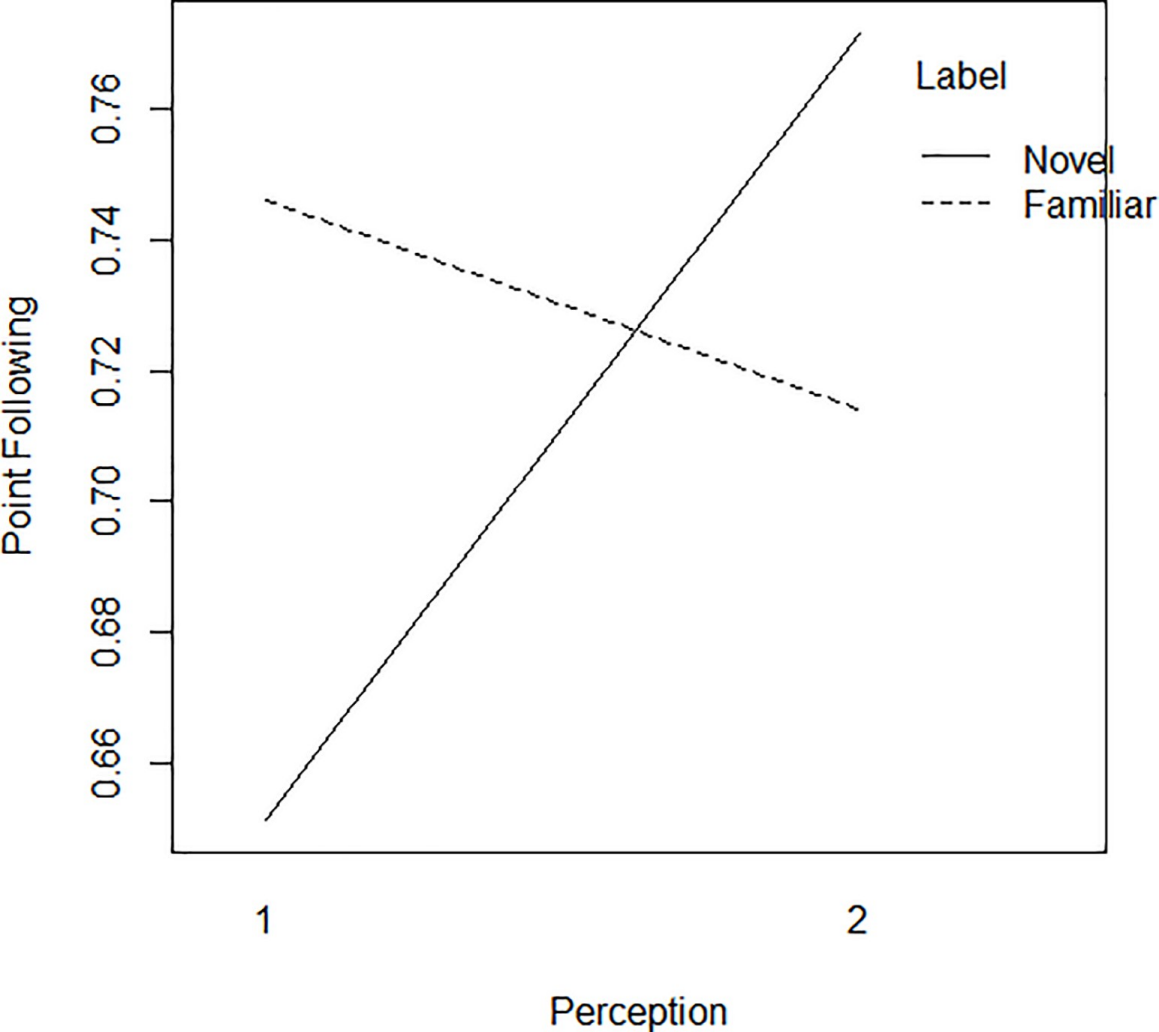
Interaction effect between label and perception. 1 = low perception, scores between 0 and 8 (*n* = 30); 2 = high perception, scores between 9 and 12 (*n* = 30).

These results showed a significant interaction between ‘label’ and ‘perception’, which indicated that the effect of ‘label’ was different for children who perceived the robot as less human-like and children who perceived it as more human-like. Surprisingly, this interaction was found across the two conditions, and thus also for the human-speaker condition. However, a three-way interaction that approached significance indicated that the ‘label’*’perception’ interaction was stronger for the robot condition than for the human-speaker condition. Specifically, as shown in Fig 3 below, children who perceived of the robot as less human-like followed pointing over labeling more often after a familiar label than after a novel label, whereas children who perceived of the robot as more human-like showed the opposite difference–albeit smaller, with more point following if a novel label rather than a familiar label was presented. Since this three-way interaction did not surpass the .05- alpha level, it should be interpreted with caution, however. Note that perception was a continuous variable in our analyses (i.e., sum scores reflecting children’s yes-answers to questions about the robot’s human-like properties, see under Analyses). An interaction between two continuous variables is difficult to plot. Therefore, our continuous perception variable was recoded into a binary ‘high’-‘low’ variable to enable visual presentation of the results in Fig 3.

## Discussion

The main aim of Study 1 was to examine whether children weighed a robot’s pointing gestures in the presence of a conflicting verbal cue differently than such cues from a human speaker. Further aims were to examine if any differences in children’s point following were affected by children’s familiarity with the verbal label and the degree to which children anthropomorphized the robot.

Our results indicated, first, that children followed the pointing gesture significantly above chance in all conditions, in keeping with previous studies showing a preference for pointing over labeling [8,14,17,18]. No difference was found in children’s reliance on non-verbal cues depending on whether a robot or a human provided these cues. This result aligns with the results by Kory Westlund et al. [21], who found no difference in children’s ability to use the non-verbal cues across a robot and a human.

Unlike in earlier studies [8,4,17], no significant differences were observed in children’s point following depending on whether pointing was pitted against a familiar or a novel label. Yet, when children’s perception of the robot as displaying human-like properties was taken into account, a significant interaction between ‘perception’ and ‘label’ emerged: Children who perceived of the robot as displaying many human-like properties relied on pointing more after hearing a novel label rather than a familiar label, while children who perceived of the robot as being less human-like relied on pointing more after hearing a familiar label rather than a novel label. Thus, only the children who perceived the robot as displaying many human-like qualities behaved similarly to the participants in earlier work with human experimenters [8,14,17]. A three-way interaction between ‘speaker’, ‘label’ and ‘perception’ showed a trend towards significance, moreover, suggesting that the differential effect of label familiarity for children varying in perception scores mainly held for the robot condition.

These findings suggest that the degree to which children weigh pointing cues as opposed to labeling cues is impacted by their perception of the robot as resembling a human. Specifically, our findings suggested that children who regarded the robot as more human-like trusted the verbal information of the robot more if it produced an existing word. When the robot uttered a novel word, however, they were reluctant to accept this is as a verbal label for the known referent, and relied on the robot’s pointing gesture instead. Children who regarded the robot as less human-like, in contrast, did not show the expected pattern, such that that they did not trust the familiar words of the robot as labels of the familiar objects more than they trusted the novel words as labels of the novel objects. This is interesting, as it suggests that children who considered the robot to be low in human-likeness may assume less common ground–in this case, shared linguistic knowledge–with the robot than children who considered it to be more human-like.

An unexpected outcome in our study was that only a subset of the children (i.e., those with high anthropomorphism scores) relied more on pointing after hearing a novel label than after hearing a familiar label, unlike in earlier studies using very similar tasks [8,14]. There are at least two possible explanations of this discrepancy in results. First, it may be due to the fact that we used images rather than physical objects, perhaps leading to a less clear-cut distinction between novel and familiar objects. A second possibility relates to the participants in the current study being older than the children in earlier studies (four- and five-year-olds vs. two- and four-year-olds [8] or two- and three-year-olds [14]). Perhaps, older children are less reluctant to assume a second referent for a familiar object than younger children, as they are more familiar with homonyms than younger children.

Children’s older age may also explain why children followed pointing less often overall than in earlier work using very similar tasks. Older children and children with larger vocabularies have been shown to rely more strongly on mutual exclusivity than younger children and children with smaller vocabularies [40,41], which has been attributed to their better knowledge of both objects and labels, which enables them to avoid lexical overlap better [42,43]. It is noteworthy, however, that, within our sample, differences in age did not have an effect on children’s pointing. Another possible explanation of why children followed pointing less often overall in our study than in previous work relates to the difference in stimuli materials. Perhaps, tapping images on the tablet screen with images fading away instead of real objects that children can manipulate and slide through a ‘chute’ as in earlier work, resulted in children being less eager to select, and more thoughtful in considering their options, which, in turn, may have affected the degree to which they followed pointing over labeling. Finally, the fact that whole hand pointing rather than index finger pointing was used, may explain why children followed pointing less strongly overall than in earlier work. Crucially, since task materials, children’s ages, and pointing manner differed between studies, it is not possible on the basis of our data to determine which factor(s) explain(s) the differences between our current results and those in earlier work.

Taken together, the results of our study provide partial support for our hypotheses. Children did not show a stronger reliance on pointing over labeling if a robot rather than a human presented the referential conflict, contrary to our prediction that children would especially rely on pointing of a robot since they would trust its verbal labels less. Also, contrary to our prediction, there was no smaller effect of label familiarity in the robot than in the human-speaker condition. What we did find, however, was that children followed pointing over labeling and that a subgroup of the children showed the expected effect of label familiarity. This effect did not involve the main effect anticipated, but an interaction, however, such that children who considered the robot as more human-like showed the predicted effect of label familiarity (i.e., more point following with a novel label than with a familiar label), whereas children who considered the robot as less human-like showed the reverse effect.

## Study 2

The aims of Study 2 were similar to those of Study 1. Specifically, we investigated whether (i) children’s reliance on eye gaze versus labeling differed between a robot and a human speaker, (ii) children showed a smaller effect of label familiarity with a robot versus a human, and (iii) children who considered the robot as human-like relied more strongly on its eye gaze than children who considered it less human-like.

## Method

### Participants

Participants were 42 monolingual Dutch children (25 girls, 60%) with an average age of 60.4 months (*SD* = 6.4, range = 50–74). Mean age was 60.7 (*SD* = 7.0) and 60.1 (*SD* = 6.4) months for boys and girls, respectively. Six additional children were tested, but not included in the final sample as they had not completed the perception questionnaire. We only recruited monolingual children, because bilingual children have been shown to rely on non-verbal cues more strongly than monolingual children [7,14,31]. Children were recruited from kindergarten classes at various schools in the Netherlands through information letters asking their parents to approve their child’s participation in the study. Written informed consent for all children was obtained from their parents prior to data collection. As in Study 1, parents filled out a questionnaire, which contained questions on home language background (to confirm that no other languages than Dutch were spoken at home) and parents’ highest attained educational level. Parents’ mean level of education was 3.73 (*SD* = 0.78) on a scale with 1 (primary school), 2 (secondary school), 3 (vocational training), 4 (higher professional education), and 5 (university) as its scale points, and averaged over children’s parents (data available for 39/42 families).

### Materials

#### Disambiguation task

**Fig 4:**
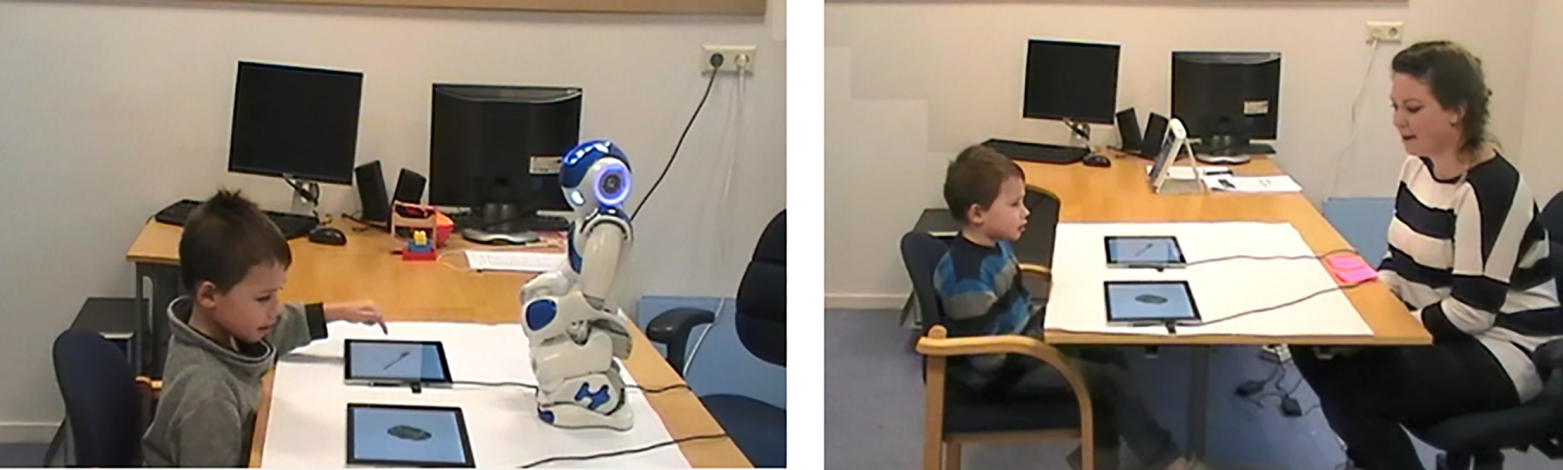
Child Performing the task contrasting eye gaze and labeling with the robot and human experimenter. (Written informed consent for publication has been obtained from the child’s parents).

The same task as in Study 1 was used, except that eye gaze instead of pointing was used as the non-verbal cue (see Fig 4). The aim of the task was to assess which cue children relied on most to solve the conflict: eye gaze or labeling. As in Study 1, two conditions were tested. First, in the ‘familiar-label condition’, the experimenter said the Dutch equivalent of the following instruction “Let’s play with the car. Tap on the car”. While producing this instruction, she gazed at the novel object. In the ‘novel-label’ condition, the experimenter said the Dutch equivalent of “Let’s play with the modi. Tap on the modi”. While producing this, she gazed at the familiar object (i.e., car). Specifically, eye gaze was as follows: The speaker looked at the child while pronouncing the Dutch equivalent of “Let’s play with the”. Then, the speaker gazed at the object while pronouncing the label for the first time (e.g., “modi”). Then, she looked back to the child while pronouncing the Dutch equivalent of “Tap on the” and back at the object again while saying the label for the second time. The speaker’s gaze to the object (including looking back to the child while saying “Tap on the”) lasted about three seconds.

As in Study 1, there was a within-subject design, with children participating in two ‘speaker’ conditions (human vs. robot) that were administered one week apart on average and two ‘label conditions’ (familiar label vs. novel label) that were administered within each session. All task materials were exactly the same as in Study 1 and the same counterbalancing procedures were applied. The same NAO robot was used.

#### Perception questionnaire

The same perception questionnaire as in Study 1 was used, with scores being calculated as the number of ‘yes’-answers.

#### Coding

Children’s responses were coded by trained assistants, as ‘gaze following’, ‘label following’, ‘both objects’, and ‘no response’. An additional assistant scored a randomly selected subset of videos of 10% of the children, yielding 100% agreement in scores.

#### Procedure

The procedure was exactly the same as in Study 1, both with respect to the administration of the task and the demonstration session of the robot prior to the first session. Also, the tasks within sessions were presented in the same order and intermixed with the same additional tasks not reported in this paper as in Study 1.

#### Analyses

A series of *t*-tests against chance level were performed on children’s gaze-following responses, as well as generalized linear mixed-effect regression analyses in R [36], using the lme4 package [37]. In the mixed-effect models, orthogonal sum-to-zero contrast coding was applied to the fixed effects [38] and the number of iterations was set to 100,000 [39].

To address our first and second research questions, a model was run with ‘speaker’ (robot vs. human) and ‘label’ (novel label vs. familiar label) as fixed-effect factors on children’s binary responses (0 = no gaze following; 1 = gaze following). Random intercepts were included for ‘subjects’, as well as a by-subject random slope for ‘speaker’. The dependent variable in this analysis was children’s gaze following (0 = no gaze following, 1 = gaze following). Responses in which children selected no image (*n* = 3, < 0.5%) or both images (*n* = 10, < 1.5%) were not taken into account. Additional models in which children’s age, gender, and presentation order (i.e., session with the robot first or last) were added as fixed-effect factors did not yield effects (except for a main effect of age such that older children followed gaze more often overall than younger children, Est. = 0.21, SE = .08, *z* = 2.57, *p* = .010) and did not change the effects for our variables of interest, and thus are not reported.

To address our final question regarding children’s perception of the robot, the same model was run, with an additional fixed-effect factor ‘perception’. This continuous variable was centered around zero and added as an interaction term with both ‘speaker’ and ‘label’. As above, additional models with the fixed-effect factors age, gender, and presentation order (whether they did the task with the robot first or last) were run, but did not yield effects (except for a main effect of age, *p* = .011) or change the results, and thus are not reported.

## Results

### Comparing children’s gaze following across conditions

Descriptive statistics for children’s gaze following are presented in Table 4 for the two speaker conditions and the two label conditions separately.

**Table 4:** Mean proportions and standard deviations for children’s gaze following after hearing a novel label or familiar label from a robot or a human.

	Novel label		Familiar label	
	M	(SD)	M	(SD)
Robot	0.21	(0.41)	0.08	(0.28)
Human	0.24	(0.43)	0.11	(0.31)

Children’s gaze following was low in all conditions, indicating that children had a strong overall preference for following the verbal cue over the gaze cue. In fact, a series of *t*-tests showed that children’s gaze following was significantly *below* chance level in all conditions (i.e., *t*(39) = -5.019, *p* < .001, *d* = 1.29 for the robot using a novel label; *t*(40) = -12.858, *p* < .001, *d* = 2.01 for the robot using a familiar label; *t*(39) = -4.286, *p* < .001, *d* = 0.68 for the human using a novel label; *t*(39) = -9.635, *p* < .001, *d* = 1.52 for the human using a familiar label).

Results of a linear mixed-effect model on children’s gaze-following responses with ‘speaker’ (robot vs. human) and ‘label’ (novel vs. familiar) as fixed effects showed a main effect of ‘label’ such that children followed eye gaze over labeling significantly more often with a novel label than with a familiar label, regardless of speaker condition (see Table 5). There was no effect of ‘speaker’ and no significant interaction between ‘speaker’ and ‘label’.

**Table 5:** Results of a linear mixed-effect model on children’s gaze following with speaker (robot vs. human) and label (novel vs. familiar) as fixed factors.

	Estimate	SE	*z*	*p*
Intercept	-5.067	1.089	-4.655	< .001
Speaker	1.783	1.504	1.186	.236
Label	2.453	0.454	5.395	< .001
Speaker*Label	-0.351	0.916	-0.383	.702

### Investigating the effect of children’s perception of the robot

Children’s mean score on the perception questionnaire was 7.4 (*SD* = 3.2). To investigate whether children’s perception was related to their gaze following across conditions, the above model was re-run, with children’s perception scores as an additional fixed-effect factor. The results of this model, presented in Table 6, showed that children relied more on gaze when a novel versus a familiar label was presented, regardless of condition. There were no effects of ‘perception’, and no significant interactions between ‘perception’ and the other factors.

**Table 6:** Results of a linear mixed-effect model on children’s gaze following with speaker (robot vs. human), label (novel vs. familiar), and perception as fixed factors.

	Estimate	SE	*z*	*p*
Intercept	-5.152	1.156	-4.458	< .001
Speaker	1.371	1.595	0.860	.390
Label	2.582	0.497	5.200	< .001
Perception	-0.150	0.229	-0.655	.513
Speaker*Label	-0.091	0.996	-0.092	.927
Speaker*Perception	0.156	0.230	0.677	.499
Label*Perception	-0.139	0.151	-0.926	.355
Speaker*Label*Perception	-0.26	0.288	-0.884	.377

### Comparing children’s reliance on pointing versus eye gaze

To compare children’s reliance on non-verbal cues as opposed to verbal cues across the two studies, we ran an additional analysis on the collapsed data from Studies 1 and 2. More precisely, we ran a linearized mixed-effect model, as above, with ‘cue type’ (pointing vs. eye gaze) as an additional fixed-effect factor. The results of this analysis showed a main effect of ‘cue type’ (Est. = 7.389, SE = 1.232, *z* = 5.996, *p* < .001) such that children followed pointing over labeling significantly more often than they followed eye gaze over labeling. The model also showed a main effect of ‘label familiarity’ such that the non-verbal cue was followed more often when it contrasted with a novel label than when it contrasted with a familiar label (Est. = 1.037, SE = 0.242, *z* = 4.293, *p* < .001). Finally, two interaction effects were found. First, a marginally significant interaction between ‘speaker’ and ‘non-verbal cue’ indicated that children followed pointing cues over labeling more often in the robot than in the human condition, while they followed eye gaze over labeling more often in the human than in the robot condition (Est. = -2.891, SE = 1.457, z = -1.973, *p* = .048). A significant interaction between ‘label familiarity’ and ‘non-verbal cue’ indicated that children followed the non-verbal cue more often after a novel label than a familiar label for eye gaze, and not pointing (Est. = -2.551, SE = 0.486, z = -5.248, *p* < .001). For the full model results, see S1 Table in the Supporting Information file.

## Discussion

In Study 2, we investigated how children weighed a robot’s eye gaze in the presence of a conflicting verbal label, as compared to their weighing of a human’s gaze and labeling cues. A further aim was to see whether the degree to which children anthropomorphized the robot predicted their reliance on the robot’s gaze.

Our results indicated that children followed eye gaze significantly below chance in all conditions. Thus, they overwhelmingly relied on the verbal label instead, irrespective of whether a robot or a human administered the task. Comparing the results across the two studies, we found that children followed pointing over labeling significantly more often than they followed eye gaze over labeling. Children’s very low reliance on eye gaze supports earlier work showing that gaze is not a strong social cue for young children [15,31,44].

Our results also showed that children relied on gaze more strongly when the gaze cue was contrasted with a novel label than with a familiar label. This effect of label familiarity aligns with earlier work and supports earlier ideas that children especially rely on non-verbal cues if they are lexically uncertain [8,14]. The degree to which children perceived of the robot as resembling a human did not predict children’s gaze following.

Most importantly, the current findings showed no clear differences in the degree to which children followed a gaze cue as opposed to a verbal cue from a robot across a robot and a human speaker. This supports earlier work by Kory Westlund et al. [21] who found no difference in children’s ability to follow gaze cues from a robot and a human either, and extends the findings of that earlier study in two ways. First, in our study, children’s reliance on eye gaze did not differ across a robot and a human in a situation in which a verbal cue was present which children could rely on instead to solve the referential conflict. Second, we found that our results were unaffected by individual differences in anthropomorphism, and thus not specific to children who saw the robot as resembling a human. Yet, a marginally significant interaction effect was found when we analyzed the collapsed data from both studies, such that children followed pointing over labeling more often in the robot than in the human condition, whereas they followed eye gaze over labeling more often in the human than in the robot condition. Possible interpretations of this result relate to the ways in which the non-verbal cues were performed by the robot. Regarding pointing, whole hand pointing was used. Perhaps, the fact that children are used to index finger pointing by humans and do not have prior experiments with pointing by robots explains why, in the current study, children tended to follow pointing more often in the robot than in the human condition. For eye gaze, in contrast, the robot’s gaze might have been too subtle for children to serve as a cue, as the robot did not have eyelids, such that its gaze was mainly instantiated by its orientation of the head rather than through a combination of head orientation and eyelid movements, as with humans. Note, however, that this interaction between ‘speaker’ and ‘cue type’ should be interpreted with caution. First, it was just below the .05-alpha level (*p* = .048). Second, it only emerged after the data from both experiments were pooled, and no effects of speaker appeared when the data were analyzed separately. Therefore, future research is needed to establish whether this difference in children’s reliance on pointing versus eye gaze across a robot and a human is robust, ideally using robots that mimic human’s pointing and gaze behaviors more closely.

Our results ran counter to our prediction that children would rely more on eye gaze of a robot than of a human in a task as the current one, as they would trust the robot’s verbal labels less. Neither did they support our prediction that children would show a smaller effect of label familiarity with a robot than with a human. Finally, our prediction that children who anthropomorphized the robot less would rely more on its eye gaze than children who anthropomorphized the robot more was not supported, since no effect of anthropomorphism was found.

## General discussion

In this paper, we investigated how children weigh the non-verbal cues (i.e., eye gaze and pointing) and verbal cues (labeling) of a robot as compared to those of a human. In two studies, children’s reliance on non-verbal cues was assessed, using disambiguation tasks in which a robot or a human presented a conflict between a non-verbal and a verbal cue. The verbal cue either involved a familiar verbal label (e.g., “car”) or an unfamiliar verbal label (e.g., “modi”).

In line with previous research with similar tasks and human experimenters [8,14,17,18], children relied more strongly on pointing than on labeling [8,14,17], and followed pointing over labeling more often than gaze over labeling [15]. Important to note in this respect is that pointing in our study was operationalized as ostensive pointing, such that it actually involved both pointing and eye gaze, to allow us to study the natural way of pointing as well as allow comparisons with earlier work [8,14]. Our results showed, furthermore, that children did not differ in their following of non-verbal versus verbal cues between a robot and a human, regardless of whether eye gaze or pointing was used. Effects of label familiarity were found in both studies. In Study 2, children relied more strongly on eye gaze when it contrasted with a novel verbal label than when it contrasted with a familiar label (both with a robot and a human). However, in Study 1, the predicted effect of label familiarity was only found for children who considered the robot as human-like, as children who considered the robot as less human-like showed the opposite effect. However, this interaction should be interpreted with caution, given that it was only slightly stronger in the robot than in the human condition.

It is an open question why the children in our study did not follow pointing more strongly over labeling overall when it contrasted with a novel label as opposed to a familiar label. In the Discussion section of Study 1, we hypothesized that this lack of an effect of label familiarity may have been due to the use of images in our task or to children’s age. However, such explanations are difficult to reconcile with the effect of label familiarity on children’s gaze following in Study 2, which used the same materials and tested similarly-aged children. Therefore, perhaps the most likely explanation of why we did not find a main effect of label familiarity on children’s point following is that a complex interplay of specific properties of our design (including whole hand pointing) and/or the sample led children to accept novel labels for familiar objects and novel referents for familiar labels equally often.

Our main finding that children did not differ in their reliance on non-verbal as opposed to verbal cues from a robot versus a human awaits further investigation. Crucially, although previous robot-assisted language learning studies have looked into the added value of robots’ use of (iconic) gestures [11] or gesturing as part of the robot’s tutoring program [10], to the best of our knowledge, no earlier studies have investigated whether children can use a robot’s eye gaze or pointing for learning. Also, future research could address in more detail how children’s anthropomorphism relates to how children interact with robots, and on children’s learning outcomes. Differences in anthropomorphism are not trivial, as they may relate to children’s trust in a robot and, in turn, to the socio-emotional relationships they may or may not establish with a robot. As such, they bear on important ethical issues not to be neglected in child-robot interaction research.

The current study has several limitations. First, no real objects were used, since the NAO robot we used could not hold objects. Since images offer fewer affordances for young children (i.e., fewer possibilities for action, see [45]), children may have been less eager in our study to respond, perhaps influencing the way in which they resolved the conflict. Second, we introduced the robot as Robin the robot to the children, thereby perhaps biasing children towards considering the robot as a human. Although this may not have been ideal, we still found substantial individual differences in children’s anthropomorphism scores, which were related to children’s point following. Finally, since the robot was not able to move its fingers independently, whole hand pointing rather than index finger pointing was used. Pointing with the index finger is more prevalent across languages and cultures [46]. However, to the best of our knowledge, most of the humanoid robots available at present cannot move their individual fingers, if they have arms, hands, and fingers at all, leaving the question of whether the same results are obtained for index finger pointing open for future research.

Despite its limitations, the current study contributes to previous research by showing that children do not differ in their reliance on two non-verbal communicative cues if these are provided by a robot instead of a human, in a context in which children could rely on a verbal cue instead. Future research is needed to investigate if they can use such cues for (language) learning, as well as how individual differences in children’s anthropomorphism may play a role.

## Supporting information

S1 Table Results of a linear mixed-effect model on children’s non-verbal following with non-verbal cue (pointing vs. gaze), speaker (robot vs. human) and label (novel vs. familiar) as fixed factors. (DOCX)
