# Supplementary material for: Children’s reliance on the non-verbal cues of a robot versus a human
Source: PLoS One. 2019 Dec 19;14(12):e0217833. doi: 10.1371/journal.pone.0217833 (PMC6922398; doi:10.1371/journal.pone.0217833)
Supplement: S1 Table — (DOCX) [file pone.0217833.s001.docx]

**Supporting Information**

**Table S1.** **Results of a Linear Mixed-Effect Model on Children’s Non-Verbal Following with Non-Verbal Cue (Pointing vs. Gaze), Speaker (Robot vs. Human) and Label (Novel vs. Familiar) as Fixed Factors**

|  | Estimate | SE | *z* | *p* |
| --- | --- | --- | --- | --- |
| Intercept | -1.370 | 0.512 | -2.676 | .007 |
| Speaker | 0.587 | 0.468 | 1.254 | .210 |
| Label | 1.037 | 0.242 | 4.293 | < .001 |
| Non-verbal cue | 7.389 | 1.232 | 5.996 | < .001 |
| Speaker*Label familiarity | -0.351 | 0.475 | -0.739 | .460 |
| Speaker*Non-verbal cue | -2.891 | 1.465 | -1.973 | .048 |
| Label familiarity*Non-verbal cue | -2.551 | 0.486 | -5.248 | < .001 |
| Speaker*Label familiarity*Non-verbal cue | 0.205 | 0.961 | 0.214 | .831 |
